# Simultaneous Heart and Kidney Transplantation: A Systematic Review and Proportional Meta-Analysis of Its Characteristics and Long-Term Variables

**DOI:** 10.3389/ti.2024.12750

**Published:** 2024-05-31

**Authors:** Natália Zaneti Sampaio, Matheus Daniel Faleiro, Laynara Vitória da Silva Vieira, Gabriele Eckerdt Lech, Sofia Wagemaker Viana, Clara Pereira Oliveira Tavares, Adela D. Mattiazzi, George W. Burke

**Affiliations:** ^1^ University of Araraquara, Araraquara, Brazil; ^2^ Federal University of Minas Gerais, Belo Horizonte, Minas Gerais, Brazil; ^3^ Federal University of Piauí, Teresina, Piauí, Brazil; ^4^ Pontifical Catholic University of Rio Grande do Sul, Porto Alegre, Rio Grande do Sul, Brazil; ^5^ Kursk State Medical University, Kursk, Kursk Oblast, Russia; ^6^ Salvador University, Salvador, Bahia, Brazil; ^7^ Leonard M. Miller School of Medicine, University of Miami, Miami, FL, United States

**Keywords:** transplant, multiorgan transplant, simultaneous heart and kidney transplantation, heart transplantation, kidney transplantation

## Abstract

Patients with end-stage heart disease who undergo a heart transplant frequently have simultaneous kidney insufficiency, therefore simultaneous heart and kidney transplantation is an option and it is necessary to understand its characteristics and long-term variables. The recipient characteristics and operative and long-term variables were assessed in a meta-analysis. A total of 781 studies were screened, and 33 were thoroughly reviewed. 15 retrospective cohort studies and 376 patients were included. The recipient’s mean age was 51.1 years (95% CI 48.52–53.67) and 84% (95% CI 80–87) were male. 71% (95% CI 59–83) of the recipients were dialysis dependent. The most common indication was ischemic cardiomyopathy [47% (95% CI 41–53)] and cardiorenal syndrome [22% (95% CI 9–35)]. Also, 33% (95% CI 20–46) of the patients presented with delayed graft function. During the mean follow-up period of 67.49 months (95% CI 45.64–89.33), simultaneous rejection episodes of both organ allografts were described in 5 cases only. Overall survival was 95% (95% CI 88–100) at 30 days, 81% (95% CI 76–86) at 1 year, 79% (95% CI 71–87) at 3, and 71% (95% CI 59–83) at 5 years. Simultaneous heart and kidney transplantation is an important option for concurrent cardiac and renal dysfunction and has acceptable rejection and survival rates.

## Graphical Abstract

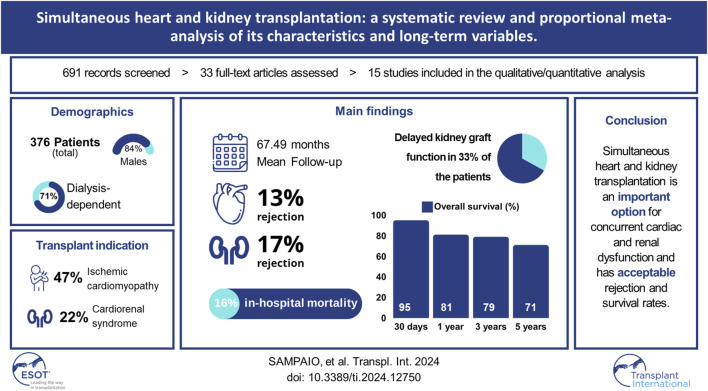

## Introduction

Patients with end-stage heart disease who undergo heart transplantation alone (HTx) frequently have simultaneous kidney insufficiency, leading to an outcome of reduced survival [1], as kidney failure is a predictor of morbidity and mortality in patients after HTx [2]. Since simultaneous heart and kidney transplantation (sHKTx) was first described in 1978 by Norman et al [3], it has become a recognized therapy for simultaneous end-stage cardiac and renal failure, with increased numbers since 2010 and representing more than 5% of the total number of HTx performed in the United States currently [4].

Indications for sHKTx are challenged by difficulties in differentiating those patients with cardiorenal syndrome without intrinsic renal disease, who could present renal recovery after HTx, from those with intrinsic advanced kidney disease who would benefit most from sHKTx [4]. Current evidence [1] supports that the simultaneous procedure is strongly recommended for heart transplantation candidates with pre-transplant renal dysfunction that leads to an estimated glomerular filtration rate (eGFR) under 30 mL/min/1.73 m [2]. Although the indications for sHKTx remain unclear, it is known that patients with simultaneous end-stage heart and renal disease who went through sHKTx have similar mortality when compared with HTx [5] and present a lower incidence of cardiac rejection [6].

The limitations of the existing sparse literature on the indications and outcomes of sHKTx highlight the critical need for studies dedicated to filling these gaps. Presently, the primary studies in this domain mainly center on retrospective analyses of the OPTN/UNOS database. However, this approach is limited as it excludes data from international centers performing this procedure. Therefore, this systematic review and meta-analysis was designed to synthesize the global evidence on the indications and outcomes of sHKTx, addressing this particular gap in the literature.

## Methods

This systematic review and meta-analysis was performed based on the Preferred Reporting Items for Systematic Reviews and Meta-Analyses guidelines (PRISMA) 2020. The study protocol was registered in AsPredicted (119472).

### Literature Search and Study Selection

We searched for relevant studies in PubMed, Embase, Lilacs, Scopus, Ovid Medline, and Web of Science updated to February 03, 2023. Two researchers (author 2 and author 3) searched works independently with a combination of the following terms: “heart kidney transplantation,” “simultaneous heart kidney transplantation” and “combined heart kidney transplantation,” and any discrepancies regarding the selection of studies were resolved by them. The reference lists of all eligible studies were reviewed for further identification of potentially relevant studies. The title and abstract of each identified publication were screened, and only publications that followed the selection criteria were fully read and included in the review.

### Selection Criteria

The inclusion criteria were as follows: 1) published clinical studies in English, Portuguese, or Spanish that investigated indications and outcomes of sHKTx patients only; 2) studies including patients that underwent sHKTx as a result of simultaneous end-stage heart disease and concomitant kidney disease; 3) studies that had reported at least one of the outcomes of interest (mean donor age, mean recipient age, recipient body mass index, left ventricular ejection fraction, pre-operative serum creatinine, heart failure etiology, renal failure etiology). The exclusion criteria were: 1) studies with incomplete or unavailable data of interest; 2) studies not involving human subjects; 3) studies that included both the staged procedure and the simultaneous procedure in the same analysis group. If multiple studies were published from the same center, with overlapping patients’ data and follow-up periods, only the most complete reports with the longest follow-up period were included for assessment.

### Data Extraction and Outcomes

The data extraction was performed with standardized processes conducted independently by two researchers (MDF, LVSV). Extracted data included study characteristics, such as title, type of study, first author, year of publication, center, study date, and the number of subjects that underwent sHKTx. The baseline demographics of the patients (donor age, recipient age, gender, cardiac and renal failure etiology, BMI, LVEF, pre-operative serum creatinine, inotrope usage, and dialysis dependency), as well as perioperative outcomes (overall allograft ischemic time, cardiac and kidney allograft ischemic times, delayed graft function, and in-hospital mortality) were also included. The assessment of warm allograft time was not feasible due to the lack of reporting of these parameters in the selected studies, although cold ischemic allograft time data was collected. The immunosuppression strategies adopted by each study were assessed and included solely in the qualitative synthesis. Five long-term outcomes (duration of follow-up period, serum creatinine at follow-up, overall, cardiac and renal rejection episodes), were assessed for quantitative synthesis. Cumulative 30-day, 1-, 3- and 5-year overall survival rates were also extracted for assessment for the study. The Newcastle-Ottawa Scale (NOS) - a tool to assess the quality of studies in meta-analyses, with a score from zero to nine, with zero being the worst outcome and nine classifying the paper as the best quality, developed by the Universities of Newcastle, Australia, and Ottawa, Canada [7] -was adopted to evaluate the quality of evidence in each included study by two researchers independently (author 1 and author 4) and they resolved any discrepancies in quality scoring.

### Statistical Analysis

A meta-analysis of proportions was conducted for the available recipient demographics and perioperative and postoperative variables with logit transformation. The R software version 3.6.0 (R Foundation for Statistical Computing, Vienna, Austria) was used for all data analysis and visualization. Heterogeneity was evaluated using the I2 test. As a guide, I2  < 25% indicated low, 25%–50% moderate, and >50% high heterogeneity [8]. If there was low or moderate statistical heterogeneity, a fixed-effect model was used. Otherwise, a random-effect model was adopted to evaluate variables with high heterogeneity. The meta-analysis was performed with the *metafor* package for R. Statistical significance was judged by *p* values under 0.05. Continuous data were estimated using mean with 95% confidence intervals (CI), and dichotomous data were reported using percentages with 95% CI. For some studies adopting median and range for parameters, estimated mean and standard deviation (SD) were obtained by adopting the formulas proposed by Hozo et al [9].

## Results

### Characteristics of the Selected Literature

**FIGURE 1:**
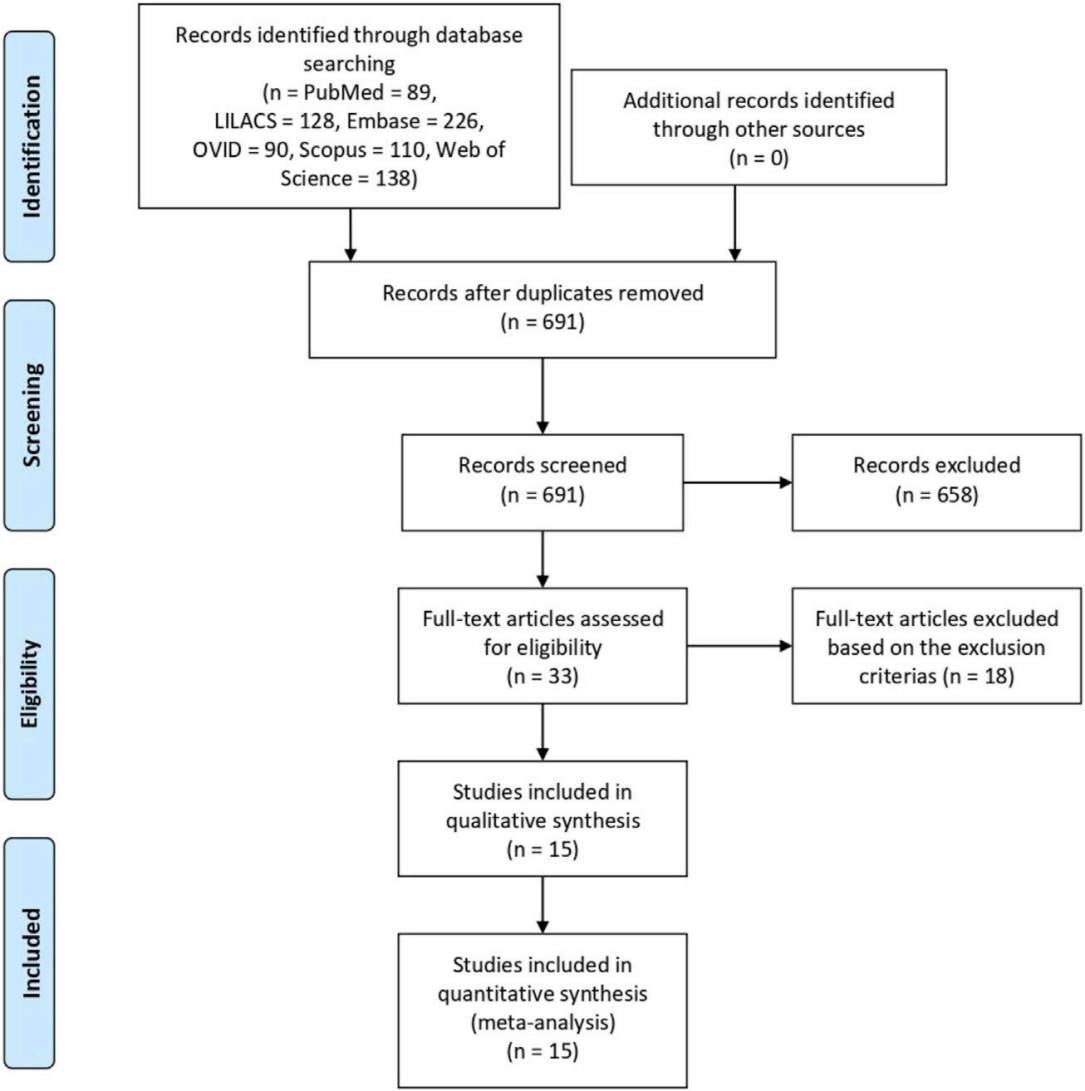
Flow diagram of literature search and study selection.

A total of 781 articles were identified based on the literature search criteria and 15 eligible papers were included in qualitative synthesis and meta-analysis [10–24], including 376 patients who underwent the procedure from 1996 to 2019. All articles were single-center retrospective studies from 15 different transplantation centers in Austria, Belgium, the United States of America, the United Kingdom, Germany, Taiwan, Italy, New Zealand, Argentina, Spain, France, and Brazil. The literature search and study characteristics description are reported in Figure 1 and Table 1, respectively. The complete results table, including heterogeneity and the number of studies used to access each pooled variable, is reported in Table A in Supplementary Material.

**TABLE 1:** Study characteristics and quality assessment.

Title	Type of study	Author and year	Institution	Country	Study date	Number of patients	New-Castle Ottawa Score (0–9)
Combined heart and kidney transplantation using a single donor: a single left’s experience with nine cases	Retrospective cohort	Kocher et al. 1998	University of Vienna	Austria	1990–1997	9	6
Combined heart-kidney transplantation: report on six cases	Retrospective cohort	Col et al. 1998	University of Louvain Medical School	Belgium	1986–1995	6	8
Simultaneous Heart and Kidney Transplantation in Patients with End-stage Heart and Renal Failure	Retrospective cohort	Leeser et al. 2001	Temple University Hospital	United States	1990–1999	13	8
Short- and long-term outcomes of combined cardiac and renal transplantation with allografts from a single donor	Retrospective cohort	Luckraz et al. 2002	Papworth Hospital	United Kingdom	1986–2002	13	8
Freedom From Graft Vessel Disease in Heart and Combined Heart- and Kidney-transplanted Patients Treated With Tacrolimus-based Immunosuppression	Retrospective cohort	Groetzner et al. 2005	Ludwig Maximilians University Hospital Grosshadern	Germany	1995–2003	13	6
Combined Heart–Kidney Transplantation: The University of Wisconsin Experience	Retrospective cohort	Hermsen et al. 2007	University of Wisconsin School of Medicine and Public Health	United States	1999–2006	19	7
Effect of simultaneous kidney transplantation on heart-transplantation outcome in recipients with preoperative renal dysfunction	Retrospective cohort	Hsu et al. 2009	National Taiwan University Hospital	Taiwan	1993–2006	13	8
Combined Heart and Kidney Transplantation: Long-Term Analysis of Renal Function and Major Adverse Events at 20 Years	Retrospective cohort	Bruschi et al. 2010	Niguarda Ca’ Granda Hospital	Italy	1989–2009	9	8
Outcomes of simultaneous heart–kidney and lung–kidney transplantations: the Australian and New Zealand experience	Retrospective cohort	Ruderman et al. 2015	4 centres across Australia and New Zealand	Australia, New Zealand	1990–2014	35	7
Combined cardiorenal transplant in heart and advanced renal disease	Retrospective cohort	Lastras et al. 2015	Hospital Universitario Fundación Favaloro	Argentina	2006–2014	20	8
Clinical Characteristics and Long-Term Outcomes of Patients Undergoing Combined Heart-Kidney Transplantation: A Single-Center Experience	Retrospective cohort	López-Sainz et al. 2015	Complejo Hospitalario Universitario A Coruña	Spain	1995–2013	22	8
Combined Heart and Kidney Transplantation: Clinical Experience in 100 Consecutive Patients	Retrospective cohort	Awad et al. 2018	Cedars-Sinai Medical Center	United States	1992–2016	100	8
Renal outcome after simultaneous heart and kidney transplantation	Retrospective cohort	Toinet et al. 2019	8 French academic lefts	France	1998–2017	73	8
Simultaneous heart-kidney transplantation results in respectable long-term outcome but a high rate of early kidney graft loss in high-risk recipients—a European single left analysis	Retrospective cohort	Beetz et al. 2021	Hannover Medical School	Germany	1987–2019	27	8
Combined Heart and Kidney Transplantation: Initial Clinical Experience	Retrospective cohort	Atik et al. 2022	Instituto de Cardiologia do Distrito Federal	Brazil	2007–2019	4	8

### Baseline Characteristics

This proportional meta-analysis included 376 patients, of whom 84% (95% CI 80–87, *p* = 0.06) were men. The mean donor age was 32.97 years (95% CI 28.21–37.73, *p* < 0.01) whereas the mean recipient age was 51.10 years (95% CI 48.52–53.67, *p* < 0.01). Regarding recipient demographics, the mean BMI was 24.42 kg/m2 (95% CI 23.42–25.41, *p* = 0.04), mean LVEF was 23.32% (95% CI 16.62–30.02, *p* < 0.01), and pre-operative serum creatinine was 4.53 mg/dL (95% CI 3.04–6.02, *p* < 0.01). Overall, 71% (95% CI 59–83, *p* < 0.01) of the patients were dialysis-dependent and 33% (95% CI 17–50, *p* = 0.02) were inotrope-dependent before transplantation.

The predominant heart failure etiology was ischemic cardiomyopathy in 47% (95% CI 41–53, *p* = 0.04) of the patients, followed by dilated cardiomyopathy in 43% of the patients, (95% CI 29–57, *p* < 0.01), and idiopathic cardiomyopathy in 28% of the patients (95% CI 20–35, *p* = 0.59). The sum of these percentages results in a value above 100% because of the variability in the assessment of each etiologies in the number of studies included, which can be verified in Table A in Supplementary Material.

Although the diagnosis methods were not registered, renal failure etiology was mostly due to cardiorenal syndrome (22% of the patients, 95% CI 9–35, *p* < 0.01), followed by glomerulonephritis (16% of the patients, 95% CI 2–30, *p* < 0.01), nephritis (14% of the patients, 95% CI 3–26, *p* = 0.01), drug-related toxicity (14% of the patients, 95% CI 9–19, *p* = 0.17), polycystic kidney disease (7% of the patients, 95% CI 4–11, *p* = 0.56), and diabetes-related (7% of the patients, 95% CI 4–11, *p* = 0.13). The sum of these percentages results in a value above 100% for the same reason mentioned before.

### Operative Variables

An immunosuppressive regimen based on induction and maintenance therapy was registered in ⅔ of the studies, and the remaining ⅓ only properly reported the use of maintenance therapy. Although it was not possible to associate the specific use of different immunosuppressive regimens with sHKTx outcome, the studies reported, as induction therapy, the use of one or more of the following: thymoglobulin, anti-thymocyte globulin, muromonab-CD3, methylprednisolone, prednisone, and basiliximab. As maintenance therapy, the studies reported the single or combined use of tacrolimus, azathioprine, mycophenolate mofetil, cyclosporine, everolimus, sirolimus, methylprednisolone, belatacept, and prednisone.

The overall cardiac allograft ischemic time was 180.46 min (95% CI 170.48–190.44, *p* = 0.01), and the overall kidney allograft ischemic time was 11.68 h (95% CI 7.87–15.48, *p* < 0.01). The studies did not define clearly how ischemic time was assessed and did not specify the difference between cold and warm ischemic time.

After transplantation, the mean ICU length of stay was 14.19 days (95% CI 1.87–26.51, *p* < 0.01). The rates of infection and sepsis were, respectively, 31% (95% CI 14–48, *p* < 0.01) and 12% (95% CI 7–17, *p* = 0.48). Delayed graft function for kidney transplantation (KTx) was presented by 33% of the patients (95% CI 20–46, *p* = 0.01), and in the hospital, mortality was 16% (95% CI 11–21, *p* = 0.17). Although not all the studies clarify the definition of how they assessed the early kidney graft function, the delayed graft function definition included requiring more than one hemodialysis. Subsequently, kidney function could be assessed by analyzing serum creatinine, eGFR, and creatinine clearance, and exclusion of any kind of rejection by biopsy. The heart graft function was reported as evaluated with the use of echography.

### Long-Term Variables

**FIGURE 2:**
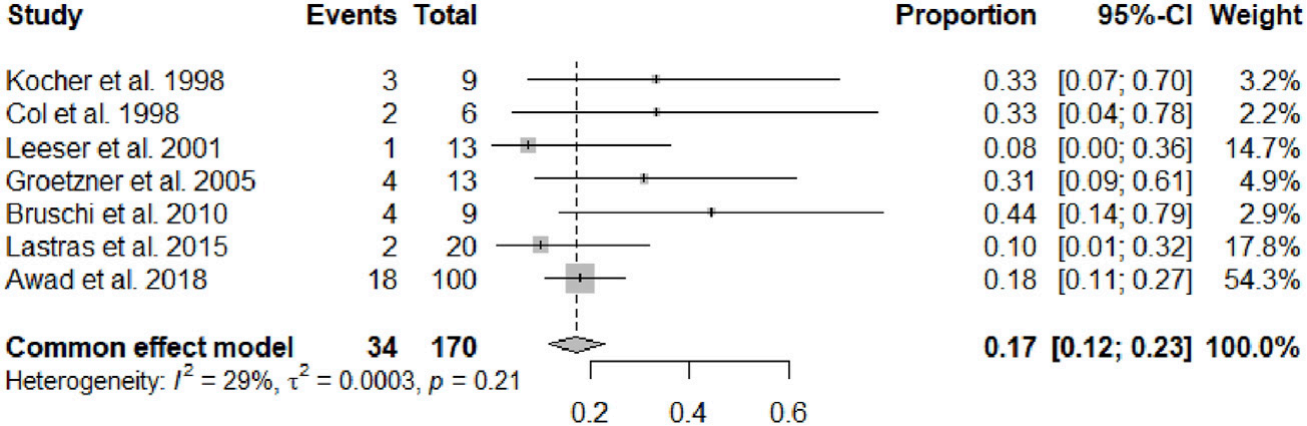
Forest plot representing the pooled occurrence of cardiac allograft rejection episode.

**FIGURE 3:**
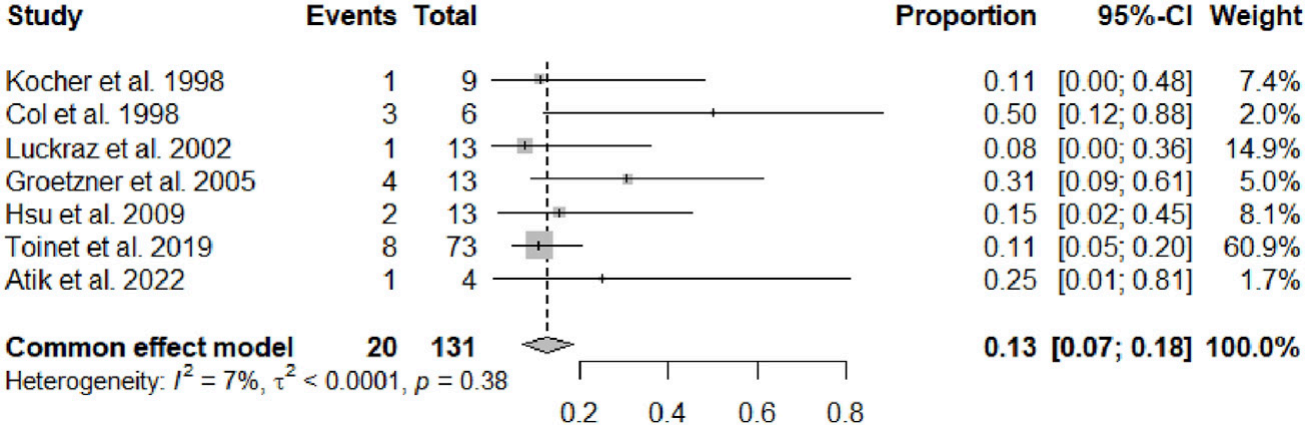
Forest plot representing the pooled occurrence of kidney allograft rejection episode.

**FIGURE 4:**
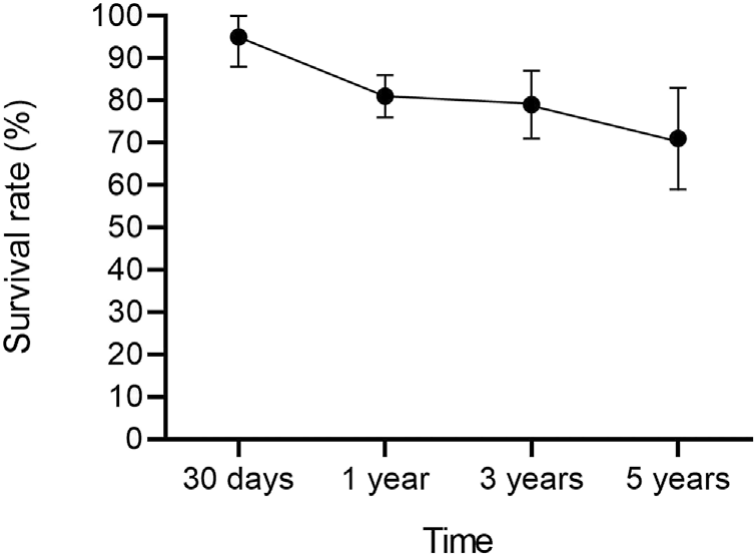
Overall survival analysis after sHKTx with 95% CI.

After a mean follow-up period of 67.49 months (95% CI 45.64–89.33, *p* < 0.01), serum creatinine was 1.50 mg/dL (95% CI 1.37–1.62, *p* = 0.16). Overall a cardiac allograft rejection episode was reported in 17% (95% CI 12–23, *p* = 0.21) of the patients (Figure 2), and a renal allograft rejection episode was also reported in 13% (95% CI 7–18, *p* = 0.38) of the patients (Figure 3). A simultaneous rejection episode of both organs’ allografts was described in 1 case by Col et al [11] and in 4 cases by Groetzner et al [14]. Allograft rejection episodes were defined after performing a renal or endomyocardial biopsy in the majority of the included studies. Overall patient survival rates of 30 days, 1-, 3-, and 5-years were, respectively: 95% (95% CI 88–100, *p* = 0.78), 81% (95% CI 76–86, *p* = 0.45), 79% (95% CI 71–87, *p* = 0.12), and 71% (95% CI 59–83, *p* < 0.01). The survival analysis is presented in Figure 4. We could not assess the specific allograft survival because of the lack of report of this information in the included studies. The patient and cardiac survival were equivalent, but the data did not describe kidney transplant survival.

## Discussion

Multi-organ transplantation is a lifesaving surgical procedure for patients with multiple organ failure and is a therapeutic option for select patients who may otherwise not survive [25, 26]. Based on the obtained results and discussion, despite its major technical and logistical challenges, we have formulated the hypothesis that this procedure delivers acceptable mortality and rejection rates. The significance of these findings lies in the fact that patients suffering from end-stage heart disease, who undergo HTx, can experience concurrent kidney insufficiency. As a consequence, their outcomes are generally unfavorable if they only receive an HTx, thereby emphasizing the need to consider KTx for these individuals, which highlights the need for studies focusing on understanding sHKTx indications and outcomes.

Although sHKTx has very scarce data in the literature, United Network for Organ Sharing (UNOS) data has shown that the number of patients on the waiting list for this procedure has progressively increased over the years [27, 28]. Our study met the recommendation of donor age to be younger than 45 years proposed by The International Society for Heart and Lung Transplantation (ISHLT) Guidelines for the Care of Heart Transplant Recipients in 2022 [29], as the mean donor age was found to be 32.97 years. However, two included studies [12, 23] described no association between donor age and better outcomes after HKTx (*p* < 0.01). In the consensus conference on sHKTx that took place on June 1, 2019, in Boston, Massachusetts, the discussion of adopting possible age cutoffs on sHKTx donors and recipients was not supported due to ethical principles [4].

Regarding the etiology of organ failure, a previous study based on the UNOS platform [30] reported ischemic cardiomyopathy (35%) and diabetes mellitus (15%) as the leading etiologies of heart and kidney failure, respectively. The results of this previous analysis are comparable with ours concerning heart failure etiology, as the main etiology for this disease was found to be ischemic cardiomyopathy in 47% of the patients included in our analysis. Although diabetes was found to be the main cause of renal failure in this previous study [30], diabetes-related kidney failure accounts for only 7% of kidney failure etiologies in our cohort, and the main etiology for this organ failure was the cardiorenal syndrome. In this syndrome, severe heart failure leads to decreased kidney perfusion and venous congestion, which consequently leads to reduced eGFR and a rise in serum creatinine [4], resulting in a cascade of feedback mechanisms that causes damage to both the organs and is associated with adverse clinical outcomes [31]. Our result is interesting because, although considered reversible, the cardiorenal syndrome was found to be the leading etiology of kidney failure in our cohort. However, regardless of the etiology of organ failure, current evidence supports the need to focus on measuring the kidney and heart function before sHKTx [15, 19, 21, 22, 32].

Pre-transplant dialysis dependence was significantly different between our analysis, which found a high prevalence of pre-transplant dialysis dependence of 71% of the patients, and the UNOS analysis [28] which registered a percentage of 40.2%. In this context, even though the dependence and the time on dialysis could not be fully assessed in the studies due to a lack of data, the pre-transplant dialysis dependence duration may be the best clinical index when determining who should be on the sHKTx transplant list [33, 34]. Patients whose hemodialysis started earlier at a higher eGFR (eGFR >10.5 mL/min per 1.73 m^) are associated with more comorbidities (hypertension and diabetes), malnutrition (serum albumin lower than 3.5 g/dL), and risk of death [31]. According to the Notice of the Organ Procurement and Transplantation Network (OPTN) Policy Change for Heart-Kidney transplant allocation of 2023 [29], candidates for transplant with CKD, eGFR less than or equal to 60 mL/min for more than 90 consecutive days, and regularly administered dialysis are acceptable for sHKTx.

In our perioperative analysis, attention was given to the allograft ischemic time in sHKTx (*p* = 0.01), as shorter renal cold ischemic times have been previously proposed as a reason for high long-term graft survival after this procedure [35, 36]. The kidney can be safely transplanted as soon as the heart function is restored, and as quickly as possible [11]. In our analysis, the kidney and heart cold ischemic time was documented in seven studies (*p* < 0.01), with the mean value of heart cold ischemic time of 183.2 min [10, 11, 15–17, 19, 22], and the mean value of kidney cold ischemic time of 11.68 h [10, 11, 15, 17, 19, 22, 23] (*p* < 0.001). It is known that the heart and kidney have different acceptable cold ischemic times without damage, but the increased time can lead to acute rejection, graft loss, and delayed graft function [37–41]. To avoid this complication, the included studies adopted different strategies to reach the lowest overall ischemic time possible, for example, donors were transferred to the same hospital as recipients to reduce heart ischemic time [16, 24]. However, these single-center studies did not assess the significance of the association between cold or warm ischemic time and sHKTx outcomes.

To prevent injury and improve graft function and surgical long-term outcomes, Machine Perfusion (MP) may be used when longer allograft kidney cold ischemic time is anticipated [42]. In our study, we could not determine the impact of MP on patients who had undergone sHKTx. It is important to recognize the many variables that are related to MP, such as the machine type and perfusion time on the machine, which can affect the transplantation outcome [42–49].

In the postoperative analysis, delayed graft function was significant in 8 studies (n = 167 patients), with approximately 33% occurrence. Although sHKTx has an outcome of acceptable delayed graft function [1], previous literature shows no significant difference in patient survival between HTx and sKHTx associated with delayed graft function [15, 19, 22, 23].

Our rejection analysis is in agreement with previous literature that reported in the early days a reduced rejection rate associated with combined transplantation [50], possibly due to an immunoprotective effect of kidney transplantation on the heart allograft [51]. This finding can be explained by the different immunosuppressive strategies that were adopted [14] and their efficacy. For that, although we were not able to analyze the relationship between the immunosuppressive strategies with sHKTx outcomes in our study, we recommend further investigation of this subject [4].

The sHKTx presented a survival rate of 81% at 1 year and 71% at 5 years in our analysis. These results are only slightly lower than the 1-year and 5-year survival rates of HTx alone, which are 84.5% and 72.5%, according to recent data from the registry of the International Society of Heart and Lung Transplantation [52]. Our 5-year survival analysis of this group of studies shows a significant variation (I2 = 69%), which the increased technical complexity of sHKTx could explain, the lack of standardized multiorgan transplantation eligibility criteria across institutions, and the lack of nationwide policies to regulate this procedure [4], that potentially leads to decreased survival rates in the current transplantation era [53].

The limitations of this study include the impossible calculation of pooled pre-transplant eGFR due to the lack of reporting of this parameter in the included studies of our cohort, which mostly reported pre-operative serum creatinine as a parameter of kidney function. For these patients who are not on dialysis, it represents a limitation because eGFR is the best overall index of kidney function [54], and the adoption of serum creatinine to estimate eGFR is not precise [55], potentially leading to the overdiagnosis of chronic kidney disease. We highlight the need for the report of eGFR in a retrospective cohort study about sHKTx, so future studies could understand how this parameter affects sHKTx outcomes.

This study has other limitations since not all the centers included in this analysis used the same patient and donor selection criteria or adopted the same sHKTx techniques and immunosuppressive regimens, so our results must be interpreted carefully. Despite a lack of granularity due to inconsistent data reports in the literature, this study stands out for its comprehensive synthesis of existing research and potential guidance to future studies and valuable insights after identifying gaps in the literature.

Also, our meta-analysis has challenges with the studies’ heterogeneity and the lack of clarity in variables such as the types of organs used. Regardless of these limitations, this is an important analysis to be conducted in the current literature to discuss HKTx indications and outcomes, which may help to guide future clinical practice. This study includes a 33-year period of literature analysis, with significant temporal and regional policy differences, consisting in limitations and unique strengths for this study, since there are clinical experiences from the 1980s up to the present time and so reflect many of the advances in both the surgical and medical management of these high-risk patients.

## Conclusion

sHKTx appears as an effective option for simultaneous end-stage cardiac and renal failure treatment, as it presents acceptable rejection and survival rates. However, further investigation is warranted to ascertain the specific patient population that would benefit the most from this procedure. We encourage future meticulous studies on this theme, with extended data reported. Additionally, global policies should be established to fortify the implementation of sHKTx and improve its outcomes. Nonetheless, it will be important to determine the physiological characteristics that may lead to renal recovery after Acute Kidney Injury (AKI) related to the cardiorenal syndrome.

## Data Availability

The original contributions presented in the study are included in the article/Supplementary Material, further inquiries can be directed to the corresponding author.
