# Supplementary material for: Simultaneous Heart and Kidney Transplantation: A Systematic Review and Proportional Meta-Analysis of Its Characteristics and Long-Term Variables
Source: Transpl Int. 2024 May 31;37:12750. doi: 10.3389/ti.2024.12750 (PMC11176494; doi:10.3389/ti.2024.12750)
Supplement: Supplementary file 1 [file DataSheet1.PDF]

Variable	Pooled value (95% CI)	N. of studies	Heterogeneity (I^2, %)	p value
Donor variables				
Age (years)	32,97 (28,21 - 37,73)	10	97	<0,01
Recipient variables				
Age (years)	51,10 (48,52 - 53,67)	14	82	<0,01
Male (%)	84 (80-87)	14	40	0,06
BMI (kg/m^2)	24,42 (23,42 - 25,41)	4	63	0,04
LVEF (%)	23,32 (16,62 - 30,02)	3	93	<0,01
Inotrope dependency (%)	33 (17 - 50)	3	76	0,02
Pre-op creatinine (mg/dL)	4,53 (3,04 - 6,02)	6	67	<0,01
Dialysis dependency (%)	71 (59 - 83)	15	89	<0,01
Heart failure etiology (%)				
Ischemic cardiomiopathy	47 (41-53)	11	48	0,04
Dilated cardiomiopathy	43 (29-57)	10	73	<0,01
Idiopatic cardiomiopathy	28 (20-35)	4	0	0,59
Renal failure etiology (%)				
Cardiorenal syndrome	22 (9 - 35)	7	71	<0,01
Glomerulonephritis	16 (2 - 30)	5	77	<0,01
Nephritis	14 (3 - 26)	6	66	0,01

Drug-related toxicity	14 (9 - 19)	6	36	0,17
Polycystic kidney disease	7 (4-11)	7	0	0,56
Diabetes related	7 (4-11)	6	41	0,13
Operative variables				
Overall ischemic time (minutes)	146,62 (87 - 206,23)	4	96	<0,01
Cardiac allograft ischemic time (minutes)	180,46 (170,48 - 190,44)	6	47	0,1
Kidney allograft ischemic time (hours)	11,68 (7,87 - 15,48)	6	97	<0,01
ICU length of stay (days)	14,19 (1,87 - 26,51)	3	94	<0,01
Infection (%)	31 (14 - 48)	11	94	<0,01
Sepsis (%)	12 (7 - 17)	8	0	0,48
Delayed graft function (%)	33 (20 - 46)	8	61	0,01
In hospital mortality (%)	16 (11 - 21)	9	32	0,17
Long-term variables				
Follow up period (months)	67,49 (45,64 - 89,33)	9	82	<0,01
Post-op creatinine (mg/dL)	1,50 (1,37 - 1,62)	4	41	0,16
Cardiac allograft rejection episode (%)	17 (12 - 23)	7	29	0,21
Renal allograft rejection episode (%)	13 (7 - 18)	7	7	0,38

Overall survival (%)				
30 days	95 (88 - 100)	6	0	0,78
1 year	81 (76 - 86)	10	0	0,45
3 years	79 (71 - 87)	6	43	0,12
5 years	71 (59 - 83)	7	69	<0,01

Table A - Results summary.
